# Hydroxychloroquine as Treatment for Inflammatory Subglottic Stenosis: A Second Successful Case

**DOI:** 10.1155/2012/754308

**Published:** 2012-11-06

**Authors:** G. Desuter, A. Gregoire, Q. Gardiner, F. Houssiau

**Affiliations:** ^1^Voice and Swallowing Clinic, Department of Otolaryngology and Head and Neck Surgery, Cliniques Universitaires Saint-Luc, Universite Catholique de Louvain, 10 Avenue Hippocrate, 1200 Brussels, Belgium; ^2^Department of Otolaryngology Head and Neck Surgery, University of Dundee, Ninewells Teaching Hospital, Dundee, UK; ^3^Department of Rheumatology, Cliniques Universitaires Saint-Luc and Universite Catholique de Louvain, Brussels, Belgium

## Abstract

Inflammatory sub-glottic stenosis is a life threatening condition that represents a therapeutic challenge. Recently, hydroxychloroquine has been suggested as one efficient medical treatment option. This report describes the second case of successful treatment of inflammatory sub-glottic stenosis using hydroxychloroquine.

## 1. Introduction

Subglottic edema is a common feature following prolonged or traumatic intubation.

This edema is part of a local inflammatory cascade causing ulceration, granulation, local devascularization and eventually chondritis of the underlying cartilaginous structures.

The end point of this process results in significant cartilaginous necrosis leading to a circumferential fibrotic stenosis covered by respiratory epithelium [1].

Some patients never reach that end point and will remain at the stage of local edema and granulation causing an inflammatory, possibly life threatening, subglottic stenosis.

These patients respond to corticosteroid treatment but over time become steroid dependent despite all the adjunctive medical treatments put in place.

Hirshoren et al. recently described a patient successfully treated with hydroxychloroquine allowing steroid weaning after 5 months of therapy [1].

We present a second case of successful treatment of steroid dependent inflammatory subglottic stenosis treated with high doses of hydroxychloroquine.

## 2. Case Report

A 56-year-old man had an emergency intubation for heart failure. He had been type 1 diabetic since the age of 20, suffered from hypercholesterolemia and hypertension, and had undergone an angioplasty of the left anterior descending artery more than 10 years before. The medical workup showed a myocardial infarction due to proximal and medial stenosis of the left anterior descending artery. An angioplasty with insertion of a drug-eluting stent was then performed. He was extubated on the 5th day allowing discharge from the intensive care unit and then spent a further week in the coronary care unit. Six weeks after hospital discharge, he was readmitted to the emergency department with acute dyspnea and stridor. 

Fiberscopic examination showed a posterior subglottic inflammatory granuloma along with circumferential subglottic edema causing hypomobility of both vocal folds fixed in adduction.

**Figure 1:**
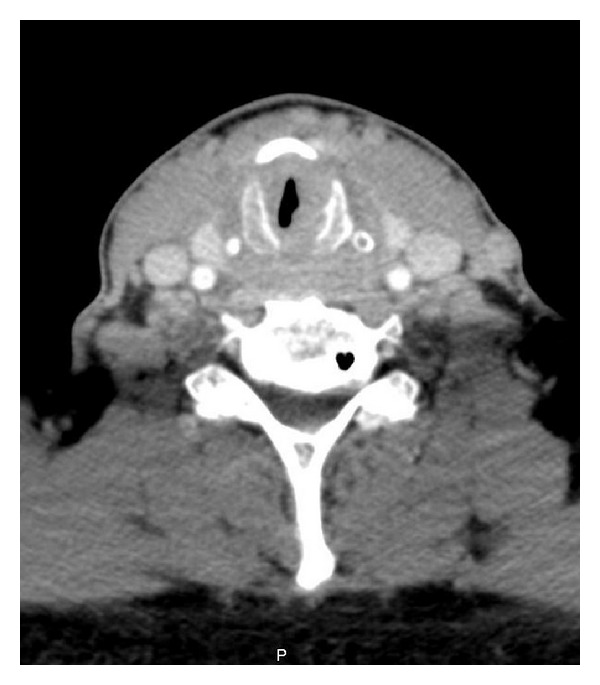
CT scan of the neck. Axial view showing subglottic edema and granuloma tissue on the left posterior commissure.

A CT scan of the neck was performed which confirmed a glottic narrowing, a thickening of the subglottic laryngeal wall with a depth of 13.5 mm causing a 41 mm^2^ subglottic stenosis (Figure 1). Cartilaginous structures of the larynx showed no sign of necrosis. This acute episode of dyspnea was treated with amoxicillin-clavulanic acid (1 g tds) and dexamethasone (1 mg/kg/day) intravenously. Oral proton pump inhibitor (pantoprazole 40 mg/day) and adrenalin inhalation was added to the treatment. Restoration of normal respiration and cessation of stridor was achieved within less than 48 h. A check CT scan and fiberscopic examination confirmed reduction in the size of the inflammatory granuloma and disappearance of the subglottic edema. The patient was discharged on a reducing dose of oral steroids. Unfortunately, symptoms reappeared after 10 days when the steroid dose had been reduced to 0.2 mg/kg/day. The same “back-and-forth” recurrences occurred several times after reduction of the oral dexamethasone dose from 0.4 mg/kg/day to 0.2 mg/kg/day.

This situation of chronic steroid use also led to challenging problems in managing his diabetes.

Ten weeks after intial intubation we performed a direct laryngoscopy under general anesthesia. This laryngoscopy allowed CO_2_ laser removal of the inflammatory granuloma, dilatation of the subglottic stenosis using Savary dilators, and a submucosal injection of steroid (dexamethasone 40 mg) directly to the affected area. 

Symptoms recurred one week after the procedure.

**Figure 2:**
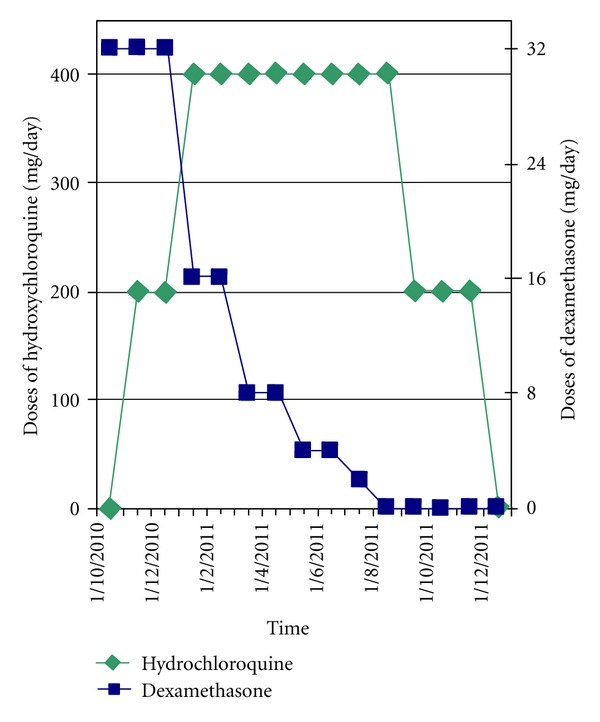
Medication dose versus time.

The patient was therefore commenced on hydroxychloroquine (Plaquenil) as reported by Hirshoren et al. [1]. After careful ophthalmological baseline examination and patient consent being obtained, treatment started at a dose of 100 mg of hydroxychloroquine (Plaquenil) twice daily combined with dexamethasone (Medrol) 32 mg/day. In the absence of contraindications the rheumatology specialists increased the dose of hydroxychloroquine to 200 mg twice daily. The patient had no recurrence of symptoms and no recurrence of the subglottic swelling at indirect laryngoscopy following the start of this treatment protocol. He was able to be weaned off dexamethasone (Medrol) after 5 months. This greatly facilitated diabetes control. The hydroxychloroquine dose was reduced to 100 mg twice daily after 7 months and stopped completely after 11 months (Figure 2).

The patient remains symptom and disease free after 9 months of follow-up. No renal, hepatic or ophthalmic side-effects of hydroxychloroquine have been observed.

## 3. Discussion

Subglottic stenosis affects the narrowest segment of the human airway, anterior and superior to the cricoid cartilage where the diameter is approximately 40 mm for men and 30 mm for women [2].

Although the duration of intubation is an important factor in causing stenosis other issues such as the size of the tube relative to tracheal diameter, frequent changing of the tube, traumatic intubation, ongoing infection, blood pressure during intubation, female gender, steroid administration, obesity, smoking history, gastric acid reflux, and an individual idiosyncratic reaction can also predispose to tracheal stenosis.

Many authors have emphasized the importance of wound healing in the pathogenesis of subglottic stenosis.

The literature and expert opinion suggest that prompt detection and rapid intervention on these new and inflamed laryngotracheal lesions may be important in preventing the development of clinically significant laryngotracheal stenosis, curable only with surgery. Likewise, the efficacy of several drugs (combinations of antibiotics and steroids, proton pump inhibitors, mitomycin B) has been shown previously when used early in the disease process [3–5].

Hirshoren et al. were the first to add hydroxychloroquine to this armamentarium based on its effects on proinflammatory agents [1]. These effects are well established in the management of lupus erythematosus and rheumatoid arthritis.

Hydroxychloroquine causes immunomodulation (blocking low affinity antigen) by means of raising lysosomal pH. In cases of long term use rare side effects have been described such as altered eye pigmentation, visual difficulties, blood disorders, liver, muscle, or renal toxicity [6].

In comparison with the previously described case, we administered a higher dose of hydroxychloroquine (up to 400 mg per day compared to 4 mg per kg per day which equated to about 280 mg/day in that particular case) and for a longer period of time (11 months versus 5 months). In the absence of drug side-effects, the rationale for having increased the dose is twofold: (a) to try to shorten the time on steroids which was leading to problems with diabetes control and (b) to try to avoid any recurrence that could potentially lead to further intubation and therefore be responsible for new mucosal damage. 

One should be aware that these two positive results were obtained with carefully selected patients. Both patients presented with subglottic stenosis at an early inflammatory stage, mostly consisting of tissue swelling and granuloma. None of them presented massive mucosal scar or cartilaginous necrosis. Likewise, both patients presented with subglottic inflammation that responded to steroid treatment. So much so, that they became steroid dependent. Steroid dose reduction to below a specific level invariably triggered a recurrence of inflammatory signs leading to subglottic lumen narrowing and consequent stridor.

## 4. Conclusion

We report the second case published in the English language literature of subglottic stenosis cured by the use of hydroxychloroquine. Further studies should assess the efficacy of hydroxychloroquine at different pathogenic stages of stenosis and/or with patients presenting with other clinical scenarios.

Furthermore, double-blind prospective randomised studies should be performed to establish: (a) when in the disease process hydroxychloroquine should be started to be effective, (b) what dose is required, and (c) for how long.
